# Integrating depth and rigor in ethnobiological and ethnomedical research

**DOI:** 10.1186/s13002-023-00643-y

**Published:** 2024-01-05

**Authors:** Ulysses Paulino Albuquerque, Romulo Romeu da Nóbrega Alves

**Affiliations:** 1 Laboratório de Ecologia e Evolução de Sistemas Socioecológicos, Departamento de Botânica, Centro de Biociências, Universidade Federal de Pernambuco (UFPE), Recife, PE 50670-901 Brazil; 2 Laboratório de Etnobiologia, Universidade Estadual da Paraíba, Avenida das Baraúnas, 351, Bairro Universitário, Campina Grande, PB 58429-500 Brazil

**Keywords:** Ethnoecology, Human ecology, Political ethnobiology

## Abstract

Ethnobiology and ethnomedicine, traditionally descriptive disciplines chronicling Indigenous People and Local Community (IPLC) practices, face the challenge of incorporating hypothesis-driven research to address contemporary issues. This paper argues for a synergistic approach where both approaches are valued for their unique contributions to understanding human–nature interactions and informing policy.

In this Debate text, we seek to address the challenge posed by the question: *Should ethnobiology and ethnomedicine more decisively foster hypothesis-driven forefront research, which can turn findings into policy and abandon more classical folkloric studies?* Although the answer may seem obvious, it has significant implications.

In response to this challenge, Łuczaj [1] argues that there is a gap between brief scientific reports and popular guidebooks, suggesting that well-documented local studies are required to bridge this void. Furthermore, he underscores the importance of incorporating the voices and anecdotes of informants into ethnobotanical research instead of relying solely on data matrices. He advocates that ethnographic studies are vital in bringing forth these voices and that approaches grounded solely in numbers often need more beauty and depth.

Reyes-García [2] argues that while hypothesis-driven research in ethnobiology and ethnomedicine is valuable for generating new knowledge, there is insufficient research to influence policymaking. Additionally, she advocates a more inclusive and collaborative approach to research involving various stakeholders, including the IPLC, to co-produce solutions for creating sustainability.

Ethnobiology and ethnomedicine have reached a crossroads where the long-standing debate between descriptive studies and hypothesis-driven research requires resolution. With their rich narrative depth, descriptive studies capture the cultural nuances often missed by quantitative analyses. In contrast, hypothesis-driven research offers structured insights to inform conservation and policy decisions. The scholarly community must transcend the dichotomy, recognizing that each approach augments the other, ensuring a robust understanding of human–environment interactions.

We concur that there are no substantial grounds to object to the importance of descriptive studies. However, disregarding hypothesis-driven science dismisses all the advances this approach can offer for advancing knowledge and the concerns of IPLC. These approaches should not be placed on opposing ends of a spectrum, and impassioned defenses based on the disqualification of those dedicated to one or the other only fragment a field [3–5] that, in our view, has not yet matured as a science. Lakatos [6] stated *that “mature science consists of research programs in which not only novel facts but, in an important sense, also novel auxiliary theories, are anticipated; mature science—unlike pedestrian trial-and-error—has ‘heuristic power’.”* Despite advances in this field, we are still progressing slowly in this direction. Most of the time, our community seems more engaged in epistemological disputes about what is or should be ethnobiology (see [7–9]).

We begin by highlighting a few points about what was mentioned earlier. Does the development of field guides, booklets, or related materials inherently ensure the preservation of Traditional Ecological Knowledge (TEK)? Are descriptive and hypothesis-driven approaches mutually exclusive? On their own, are any of these approaches capable of influencing policymaking? We tend to answer all three questions in the negative.

In the case of the first question, preserving knowledge goes beyond simply documenting wisdom and requires researchers to engage in actions to empower the IPLC. Descriptive studies are at the heart of both ethnobiology and ethnomedicine. Oral traditions, rituals, and ancestral practices are critical to understanding the intricate interrelations between humans and the natural world. Furthermore, they showcase various cultures' belief systems, philosophies, and worldviews.

Descriptive studies have documented these traditions, capturing the essence of these cultures, often in the face of extinction threats from modernization and globalization. When reporting these knowledge systems, ethnobiology and ethnomedicine play pivotal roles in preserving the world's cultural richness and ensuring that such wisdom does not vanish [10]. Recognizing these knowledge systems also serves as a source of empowerment for IPLC. Valuing their knowledge and practices underscores the notion that their traditions are as valuable as any other modern and scientific form. However, preserving this knowledge demands more than just documentation by scientists and academics. Without deeper engagement of researchers with IPLC, this documentation may evolve into a modern form of colonialism. This raises questions: Preserve for what, why, and for whom?

Although there is a scope for descriptive research in these fields, it must be conducted rigorously and meaningfully. There is an urgent and undeniable need for hypothesis-driven studies in the area. We live in a complex and rapidly changing world where our challenges are increasingly multifaceted and interconnected. We require a methodological and empirical research approach to address these challenges effectively. As the world faces unprecedented challenges, from health crises to climate change, it is imperative to align scientific investigations with these practical and pressing issues.

This does not imply that we should forsake descriptive research in favor of something more “advanced.” Instead, we strove to make this study as comprehensive and informative as possible. Through high-quality descriptive analysis, we can lay a firm foundation for more hypothesis-driven inquiries. Regardless of the approach, we advocate for high-quality, rigorous, and meaningful research. At its core, science is a tool that should be used to understand the world. Directing this tool precisely makes us more adept at finding tangible solutions to these persistent challenges. Thus, we imply that the focus of ethnobiology as a plural science [11] is more than just documenting TEK.

Exploring the relationship between nature and humans can be conducted by researchers from various disciplines, each focusing on a particular subject matter with their own body of knowledge, theories, and methods. These researchers have introduced unique perspectives, methodologies, and tools into the study of ethnobiology. Understanding ethnobiology as an interdisciplinary science poses many epistemological and theoretical challenges in this context. Still, they are surmountable if academic science practitioners engage in constructive dialog and accept that there are different approaches to studying phenomena [12].

In broad terms, we advocate for research to adhere to the highest standards specific to the investigation, regardless of the assigned label. It is equally crucial to scrutinize each study through this lens. For instance, expecting an ethnographic approach in a study not intended to be qualitative but rather hypothesis-driven, or vice versa, needs more internal consistency. This perspective is reinforced by the argument put forth by Casedevall and Ferric [13], asserting that descriptive and hypothesis-driven science should be viewed not as opposing forces but as collaborative contributors to the advancement of knowledge. They propose that considering the common practice of categorizing science using terms like “descriptive,” “mechanistic,” “hypothesis-driven,” or “discovery-driven,” and the potential for these labels to carry either positive or negative connotations with significant consequences, it is essential to contemplate the meanings and intentions behind such terms carefully.

In this regard, the researcher and their project determine whether to decipher the discourse in ethnographic narratives or focus on spreadsheets, equations, and statistics. In other words, the beauty of ethnobiology lies in what we can unveil from the observational and descriptive elements of phenomena.

Ethnobiology and ethnomedicine, grounded in top-tier research, have the potential to inform policies in critical areas ranging from the conservation of biodiversity to public health. However, descriptive research must become more rigorous and sophisticated for this potential to be fully realized. A compelling descriptive study in ethnobiology should be anchored in the best understanding of qualitative research and the theoretical background underpinning these methodologies. It is imperative to understand that purely descriptive studies can only merely catalog or list elements by delving deeply into their interconnectedness, symbolism, and functions within the community. The depth and breadth of the narrative, the authenticity of the voices captured, and the richness of the context set apart exemplary descriptive studies.

***

To bolster the rigor and relevance of ethnobiological research, journals should consider establishing a "checklist" section dedicated solely to publishing lists of species derived from such descriptive ethnobiological studies. However, the criteria should be stringent to ensure the lists are derived using the best available methods. In this sense, biologists with expertise in plants (botanists) and animals (zoologists) play a crucial role in identifying and documenting the biodiversity utilized by different cultures. This will serve as a documentation of biodiversity as perceived and used by communities and as a foundation for future research.

Also, ethnobiological researchers should explore the broader implications of their studies to provide reflections, recommendations, and implications for their research, allowing for translating academic findings into actionable policy directives. This could appear as a section in the journals of our field as a “policy brief.” Such a section would bridge the gap between academic and policy realms, ensuring that the rich insights gained from ethnobiological research can be channeled into impactful policies.

The mission to preserve biological and cultural diversity is value-driven and implies urgency, considering that the global biodiversity crisis is caused primarily by human activities. This demonstrates that ethnobiology can make valuable contributions to preserving biocultural diversity. In this context, both descriptive studies and hypothesis-driven science play essential roles (see [14]). This combination is crucial for advancing our understanding of the intricate relationships between human cultures and their environments, increasing the scientific rigor of studies, and consolidating ethnobiology as a scientific field.

## Data Availability

Not applicable.

## Abbreviations

IPLC: Indigenous People and Local Community
TEK: Traditional Ecological Knowledge

## References

[1] Łuczaj Ł. Descriptive ethnobotanical studies are needed for the rescue operation of documenting traditional knowledge. J Ethnobiol Ethnomed. 2023;19:37. 10.1186/s13002-023-00604-5. 37679801 10.1186/s13002-023-00604-5 PMC10486101

[2] Reyes-García V. Beyond artificial academic debates: for a diverse, inclusive, and impactful ethnobiology and ethnomedicine. J Ethnobiol Ethnomed. 2023;19:36. 10.1186/s13002-023-00611-6. 37679793 10.1186/s13002-023-00611-6 PMC10486112

[3] Albuquerque UP. How to become an ethnobiologist: against the cultural monopoly. Ethnobot Res Appl. 2022;24:1–8. 10.32859/era.24.6.1-8.

[4] Albuquerque UP. What is the métier of ethnobiology, or why should this science be busy? Ethnobot Res Appl. 2022;24:1–7.

[5] Ludwig D, El-Hani CN. Philosophy of ethnobiology: understanding knowledge integration and its limitations. J Ethnobiol. 2020;40:3–20. 10.2993/0278-0771-40.1.3.

[6] Lakatos I. Falsification and the methodology of scientific research programmes. In: Lakatos I, Musgrave A, editors. Criticism and the growth of knowledge. Proceedings of the international colloquium in the philosophy of science, London, 1965. Cambridge: Cambridge University Press; 1970. p. 91–196. 10.1017/CBO9781139171434.009.

[7] Ferreira Júnior WS, Albuquerque UP, Medeiros PM. Reflections on the use of hypotheses in ethnobotany: a response to Leonti et al. (2020). Ethnobot Res Appl. 2021;21:1–5. 10.32859/era.21.18.1-5.

[8] Leonti M, Casu L, Oliveira Martins DT, Rodrigues E, Benítez G. Ecological theories and major hypotheses in ethnobotany: their relevance for ethnopharmacology and pharmacognosy in the context of historical data. Rev Bras Framacog. 2020;30:451–66.

[9] Albuquerque UP, Ferreira Júnior WS. Hypothesis testing in ethnobotany: 30 years after Phillips & Gentry’s seminal work. Ethnobiol Conserv. 2023. 10.15451/ec2023-06-12.14-1-3.

[10] Turner NJ, Cuerrier A, Joseph L. Well grounded: indigenous peoples’ knowledge, ethnobiology, and sustainability. People Nat. 2022;4:627–51. 10.1002/pan3.10321.

[11] Nieves Delgado A, Ludwig D, El-Hani C. Pluralist ethnobiology: between philosophical reflection and transdisciplinary action. J Ethnobiol. 2023. 10.1177/02780771231194774.

[12] Albuquerque UP, Jacob MCM, Alves RRN. Celebrating the 10th anniversary of ethnobiology and conservation. Ethnobiol Conserv. 2022;11.

[13] Casadevall A, Ferric F. Descriptive and hypothesis-driven research. Microbe Mag. 2009;4:207–207.

[14] Gonçalves-Souza T, Provete DB, Garey MV, da Silva FR, Albuquerque UP. Going back to basics: how to master the art of making scientifically sound questions. In: Albuquerque U, de Lucena R, Cruz da Cunha L, Alves R, editors. Methods and techniques in ethnobiology and ethnoecology . New York, NY: Springer Protocols Handbooks. Humana Press. 10.1007/978-1-4939-8919-5_7.

